# Epidemiology and Economic Burden of Puumala Virus Infection in Styria, Austria, 2003 to 2024

**DOI:** 10.1093/ofid/ofag607

**Published:** 2026-09-26

**Authors:** Stefan Hatzl, Christina Geiger, Lisa Kriegl, Markus Keldorfer, Gernot Schilcher, Florian Krammer, Robert Krause

**Affiliations:** Department of Internal Medicine, Intensive Care Unit, Medical University of Graz, Graz, Austria; BioTechMed Graz, Graz, Austria; Division of Infectious Diseases, Department of Internal Medicine, Medical University of Graz, Graz, Austria; BioTechMed Graz, Graz, Austria; Division of Infectious Diseases, Department of Internal Medicine, Medical University of Graz, Graz, Austria; Department of Children and Adolescent Medicine, Pediatric Intensive Care Unit, Medical University of Graz, Graz, Austria; Department of Internal Medicine, LKH Südsteiermark–Standort Wagna, Wagna, Austria; Department of Microbiology, Icahn School of Medicine at Mount Sinai, New York, New York, USA; Center for Vaccine Research and Pandemic Preparedness (C-VaRPP), Icahn School of Medicine at Mount Sinai, New York, New York, USA; Department of Pathology, Molecular and Cell-Based Medicine, Icahn School of Medicine at Mount Sinai, New York, New York, USA; Ignaz Semmelweis Institute, Interuniversity Institute for Infection Research, Medical University of Vienna, Vienna, Austria; BioTechMed Graz, Graz, Austria; Division of Infectious Diseases, Department of Internal Medicine, Medical University of Graz, Graz, Austria

**Keywords:** Austria, economic burden, healthcare costs, *Puumala virus*, PUUV

## Abstract

**Background:**

Hantavirus infections cause hemorrhagic fever with renal syndrome and impose a potential burden on healthcare systems. However, comprehensive economic data remain scarce. We quantified the inpatient economic burden of hospitalized patients with Puumala virus (PUUV) infections in Styria, Austria’s primary endemic region, to identify hospitalization costs and early admission predictors of costs.

**Methods:**

This retrospective cost-of-illness study analyzed 1049 serologically confirmed PUUV hospitalizations across 20 public hospitals in Styria, Austria between September 2003 and March 2024. Costs were estimated using the Austrian performance-oriented hospital financing (Leistungsorientierte Krankenanstaltenfinanzierung) diagnosis-related group system, standardized to 2024-equivalent Euros using consumer price index deflation. Admission predictors of cost were assessed using multivariable γ-generalized linear modeling with multiple imputations. Temporal trends in length of stay were analyzed using weighted segmented linear regression.

**Results:**

Cumulative inpatient cost was ∼€10.3 million (mean per-case: €9788) for the entire study period of 21 years. Three epidemic years (2012, 2019, and 2021) accounted for 53.6% of cases and over €5.2 million in expenditure, with peak annual costs reaching €1.93 million (0.274% of total Styrian inpatient expenditure) in 2012. Prolonged activated partial thromboplastin time (aPTT; cost-ratio 1.024 per second, 95% CI 1.019–1.030) and older age (CR 1.004 per year, 95% CI 1.001–1.008) were independent admission-level predictors of higher cost. Median hospital length of stay declined by ∼50% over the study period.

**Conclusions:**

PUUV hospitalizations impose a sharply episodic but recurrent economic burden on regional healthcare systems. Admission aPTT and age independently predicted cost, but clinical outcomes were not modeled and these variables do not constitute a triage tool. Epidemic preparedness strategies are warranted.

## Teaser

*Puumala* hantavirus hospitalizations cost €10.3 million over 22 years across 20 Styrian hospitals, with costs concentrated in 3 epidemic years. Admission aPTT and age independently predicted higher costs. Length of stay halved over the study period, reflecting improved clinical management.

Hantaviruses are enveloped, negative-sense, single-stranded RNA viruses belonging to the genus *Orthohantavirus* (family *Hantaviridae*, order *Bunyavirales*) [1, 2]. In Europe, and particularly in Austria, the main species causing human disease are Puumala virus (PUUV) and Dobrava-Belgrade virus (DOBV), while Saaremaa virus and, more rarely, Seoul virus (SEOV) and Tula virus have also been detected in human infections. PUUV is the predominant cause of disease and typically leads to nephropathia epidemica, a milder form of hemorrhagic fever with renal syndrome (HFRS). In contrast, DOBV is associated with more severe clinical courses and higher case fatality rates, particularly in southeastern and central Europe [1, 3]. Over the past 2 decades, hantavirus infections have increased across Europe, with more than 10 000 HFRS cases reported annually and a clear upward trend. This rise is attributed to improved recognition and diagnostics, environmental and climate changes, and fluctuations in rodent populations that serve as viral reservoirs. Epidemic peaks occur every 2–4 years, often in localized hotspots, and the geographic range of the virus has expanded in recent years [4]. Old World hantaviruses, including HTNV, DOBV, PUUV, and SEOV, cause HFRS, a disease characterized by endothelial dysfunction, increased vascular permeability, thrombocytopenia, and coagulation abnormalities. Following an incubation period of 2–6 weeks, patients typically present with abrupt fever, headache, myalgia, abdominal and back pain, and gastrointestinal symptoms. Severe cases may progress to hypotension, shock, and acute kidney injury, sometimes requiring renal replacement therapy. Mortality varies by virus species, ranging from 1% for PUUV to 5%–15% for HTNV and DOBV [1, 2]. In Austria, Southeastern regions, particularly Styria, represent the main endemic areas, with recurrent clusters of PUUV-associated nephropathia epidemica. Molecular analyses of viral strains from rodents and patients support the presence of distinct local genotypes in these regions [5, 6]. Beyond its clinical relevance, hantavirus disease imposes a considerable burden on healthcare systems. Management frequently requires hospitalization for 5–14 days, and severe cases may necessitate intensive care and dialysis. Approximately 5–7% of hospitalized PUUV cases and up to 48% of DOBV cases require renal replacement therapy, substantially increasing resource utilization. The need for advanced supportive measures further amplifies direct medical costs [2, 7, 8]. Despite the growing incidence and the potentially high resource demand, data on the direct healthcare costs of hantavirus infections remain scarce. Existing literature primarily addresses epidemiology and clinical outcomes, while comprehensive economic evaluations are largely lacking. Consequently, robust cost analyses are essential to quantify the financial impact on healthcare systems and to inform resource allocation, preparedness strategies, and public health decision-making [1, 2, 7].

## METHODS

### Study Setting in Data Source

This economic burden analysis was a prespecified top-down costing study of a retrospective multicenter cohort of PUUV infections across 20 public hospitals in the Styrian Krankenanstalten Gesellschaft (KAGes) network, covering September 2003 to March 2024. Case ascertainment began in September 2003, when retrievable electronic hospital records start in the KAGes network, and ended with database closure in March 2024, so that both calendar years are partial by design (Supplementary Appendix A3). The KAGes healthcare network provides nearly all public inpatient care in Styria, ensuring comprehensive coverage. Clinical and laboratory data were extracted from electronic patient records using a standardized case report form and managed in REDCap [9, 10]. Collected variables included demographics, presenting symptoms, comorbidities, and laboratory values within 24 hours of admission. Eligible patients had serologically confirmed PUUV infection and compatible clinical syndromes; those without serological confirmation or sufficient follow-up were excluded [6]. The study was approved by the local ethics committee (approval no. 33-329 ex 20/21) and conducted per the Declaration of Helsinki, with informed consent waived due to its retrospective design. This economic burden analysis is reported in accordance with the Consolidated Health Economic Evaluation Reporting Standards (CHEERS 2022) checklist where applicable to a cost-of-illness study design [11] (Supplementary Figure 1).

### Austrian Hospital Reimbursement System

Austrian public hospital financing is governed by the Performance-Oriented Hospital Financing (Leistungsorientierte Krankenanstaltenfinanzierung, LKF) system, a federal diagnosis-related group (DRG) framework implemented locally via state health funds (Landesgesundheitsfonds). Each inpatient episode generates a case-level point score composed of a procedure component (Leistungskomponente) for diagnostic and therapeutic interventions and a Per-Diem component (Tageskomponente) scaled to length of stay (LOS). Points are converted to Euros using state-specific tariffs. Intensive care unit (ICU) admissions receive additional daily supplements reflecting higher resource use. Fund hospitals are financed collectively rather than billed to the individual patient: statutory social health insurance contributions and public tax revenue are pooled in the state health fund, so that the costs reported here fall on the public payer rather than on patients or on individual hospitals. LKF points measure reimbursed resource utilization and not clinical severity, and laboratory tests, including serial coagulation monitoring, generate no separate points (Supplementary Appendix A1 and A2). The LKF model undergoes periodic revision, but DRG assignment and point weights for PUUV hospitalizations remained stable from 2003 to 2024, ensuring valid longitudinal cost comparisons. For more details on the LKF system see the Supplementary Methods.

### Inflation Adjustment and Cost Standardization

All costs are expressed in 2024-equivalent Euros to enable valid comparison across the 22-year study period. Patient-level LKF point scores were multiplied by the 2024 tariff (€2.16/point) and scaled by the ratio of the year-specific Austrian consumer price index (Verbraucherpreisindex; base 2020 = 100) to the 2024 index:


$$\mathrm{Cost}2024=\mathrm{LKF}\;\mathrm{Points}\;\times \text{€}\;2.16\;\times \;\frac{\mathrm{CPI}2024}{\mathrm{CPI}\;\mathrm{year}}$$

This method accounts for inflation while the confirmed stability of DRG assignments ensures the changes that reflect true differences in resource use. As a sensitivity analysis, costs were also deflated using the Organization for Economic Cooperation and Development (OECD) Health Expenditure Price Deflator, which slightly lowers early period estimates.

### Proportion of Styrian Inpatient Healthcare Expenditure

To contextualize the economic burden of PUUV, annual hospitalization costs (in 2024-equivalent Euros) were expressed as a proportion of total Styrian inpatient LKF expenditure Budget data for 2006–2024, which were obtained from KAGes and Styrian Health Fond (Gesundheitsfonds Steiermark) reports. Consolidated Styrian inpatient expenditure has been reported by the Gesundheitsfonds Steiermark only since the state health funds were established in the 2005 Austrian health care reform, so the proportional analysis covers 2006–2024 only (Supplementary Appendix A4).

### Statistical Analysis

All analyses were performed in R (version 4.4.1 R Foundation for Statistical Computing, Vienna, Austria) using key packages: mice for multiple imputation, segmented for breakpoint analysis, and ggplot2 for visualization. A two-sided significance threshold of *P* < .05 was applied.

Annual case counts, proportions requiring ICU admission or renal replacement therapy, and median hospital LOS and interquartile range (IQR) were summarized per year. Partial-year data for 2003 and 2024 were interpreted accordingly. Total costs were decomposed into base DRG and ICU daily supplement components to quantify the costs attributable to intensive care.

Admission-level predictors of 2024-equivalent LKF costs were assessed using a generalized linear model (GLM) with Gamma family and log link, suitable for strictly positive, right-skewed costs. Variables with >15% missing data were excluded; remaining missing values were handled using multiple imputation by chained equations (MICE) with 20 datasets. Models were fitted per dataset and pooled using Rubin's rules, with results reported as cost ratios (CR) and 95% confidence intervals.

Temporal trends in median LOS were analyzed using weighted segmented linear regression, with year weights proportional to case counts. Models with up to 2 breakpoints were evaluated; the best-fitting model was selected. Breakpoint locations were reported with 95% confidence intervals derived from 100 bootstrap replicates.

## RESULTS

### Cohort Description

We identified 1049 patients with confirmed PUUV infection requiring inpatient admission across 20 KAGes public hospitals in Styria, Austria, between September 2003 and March 2024 (Styria Hantanet retrospective surveillance program). As the KAGes network provides virtually all public inpatient care in the region, this cohort represents near-complete population-level capture of hospitalized hantavirus disease in Austria's primary endemic area. The median age was 46.7 years (IQR 34.4–57.3) and 295 patients (28.1%) were female. Patients had few comorbidities; arterial hypertension was present in 206 patients (19.6%), while other comorbidities occurred in fewer than 5%. At admission, fever >38.5°C was reported in 978 patients (93.2%) and headache in 676 (64.4%); 541 (51.8%) reported prior rodent exposure. Outcomes included 8 hantavirus-related deaths (0.8%), ICU admission in 100 patients (9.6%), and renal replacement therapy in 78 (7.4%) (Table 1; Supplementary Figure 2).

**Table 1.** Baseline Characteristics

Variable	Overall (n = 1049)	Missing
Demographics and baseline		
Age (years)	46.7 [34.4, 57.3]	
Sex		
Male	754 (71.9%)	
Female	295 (28.1%)	
Height (cm)	175.0 [170.0, 180.0]	29.6%
Weight (kg)	79.0 [70.0, 86.0]	24.4%
Comorbidities		
Heart failure	27 (2.6%)	
Diabetes mellitus	51 (4.9%)	
Arterial hypertension	206 (19.6%)	
Atrial fibrillation	34 (3.2%)	
Coronary artery disease	25 (2.4%)	
Thromboembolic disease	29 (2.8%)	
Chronic kidney disease	20 (1.9%)	
COPD	12 (1.1%)	
Other lung disease	19 (1.8%)	
Vascular disease	21 (2.0%)	
Dementia	1 (0.1%)	
Prior malignancy (cured)	13 (1.2%)	
Active malignancy	12 (1.1%)	
Organ/stem cell transplant	3 (0.3%)	
Immunosuppressive therapy	7 (0.7%)	
Symptoms at presentation		
Temperature at admission (°C)	39.0 [38.7, 39.8]	22.8%
Fever	978 (93.2%)	
Headache	676 (64.4%)	
Eye pain	48 (4.6%)	
Dyspnea	116 (11.1%)	
Diarrhea	180 (17.2%)	
Abdominal cramps	287 (27.4%)	
Flank/kidney pain	270 (25.7%)	
Blurred vision	161 (15.3%)	
Rodent exposure	541 (51.8%)	
Hematology		
Leukocytes (G/L)	9.0 [6.8, 11.9]	
Neutrophils (G/L)	6.1 [4.5, 8.5]	7.3%
Lymphocytes (G/L)	1.4 [1.0, 2.0]	9%
Monocytes (G/L)	1.0 [0.7, 1.4]	7.5%
Eosinophils (G/L)	0.2 [0.1, 0.3]	7.5%
Hemoglobin (g/dL)	14.9 [13.7, 16.2]	
Platelets (G/L)	78.0 [54.0, 111.0]	
Renal function		
Creatinine (mg/dL)	1.5 [1.0, 2.8]	
eGFR (mL/min/1.73 m^2^)	51.9 [24.8, 79.9]	0.5%
Urea (mg/dL)	51.0 [31.0, 86.0]	0.6%
Inflammatory markers		
CRP (mg/L)	67.0 [37.1, 111.0]	
IL-6 (pg/mL)	53.0 [29.7, 99.0]	95.5%
Procalcitonin (ng/mL)	1.2 [0.5, 2.6]	71.5%
LDH (U/L)	261.0 [218.0, 325.8]	8.3%
Liver function		
Bilirubin total (mg/dL)	0.6 [0.4, 0.7]	6.1%
GGT (U/L)	39.0 [25.0, 68.5]	1.3%
AST (U/L)	37.0 [27.0, 55.0]	1.2%
ALT (U/L)	38.0 [25.0, 61.0]	1.1%
Cardiac markers		
NT-proBNP (pg/mL)	368.5 [103.2, 1618.5]	83%
Troponin T	3.0 [3.0, 8.2]	60.7%
Coagulation		
Prothrombin time (%)	94.0 [84.0, 104.0]	5.2%
aPTT (sec)	34.9 [31.0, 40.0]	8.4%
D-Dimer (mg/L)	3.7 [1.6, 7.1]	79.4%
Antithrombin III (%)	85.0 [73.0, 98.5]	84.5%
Fibrinogen (mg/dL)	443.5 [368.0, 538.0]	70.4%
Other laboratory		
Albumin (g/dL)	3.1 [2.7, 3.6]	
Ferritin (ng/mL)	840.0 [413.0, 1735.0]	55.3%
Triglycerides (mg/dL)	254.0 [169.0, 364.0]	45%
Outcomes		
Hanta-related death	8 (0.8%)	
ICU admission	100 (9.6%)	
Renal replacement therapy	78 (7.4%)	

Abbreviations: ALT, alanine aminotransferase; aPTT, activated partial thromboplastin time; AST, aspartate aminotransferase; CRP, C-reactive protein; eGFR, estimated glomerular filtration rate; GGT, gamma-glutamyltransferase; ICU, intensive care unit; IL-6, interleukin-6; IQR, interquartile range; LDH, lactate dehydrogenase; NT-proBNP, N-terminal pro-B-type natriuretic peptide.

Data are presented as median [interquartile range] for continuous variables and as n (%) for categorical variables. The “missing” column indicates the proportion of patients with missing values for the respective variable; variables with complete data are left blank.

### Case Dynamics and Epidemic Patterns

Annual PUUV hospitalizations exhibited pronounced interannual variability (Figure 1). Three major epidemic years were identified: 2012 (n = 215 patients with PUUV—infections, the largest outbreak), 2019 (n = 188), and 2021 (n = 159). In contrast, few cases were identified in 2003 and 2024 (1–3 cases), as these calendar years were only partially covered by the study period (September to December 2003 and January to March 2024, respectively).

**Figure 1.**
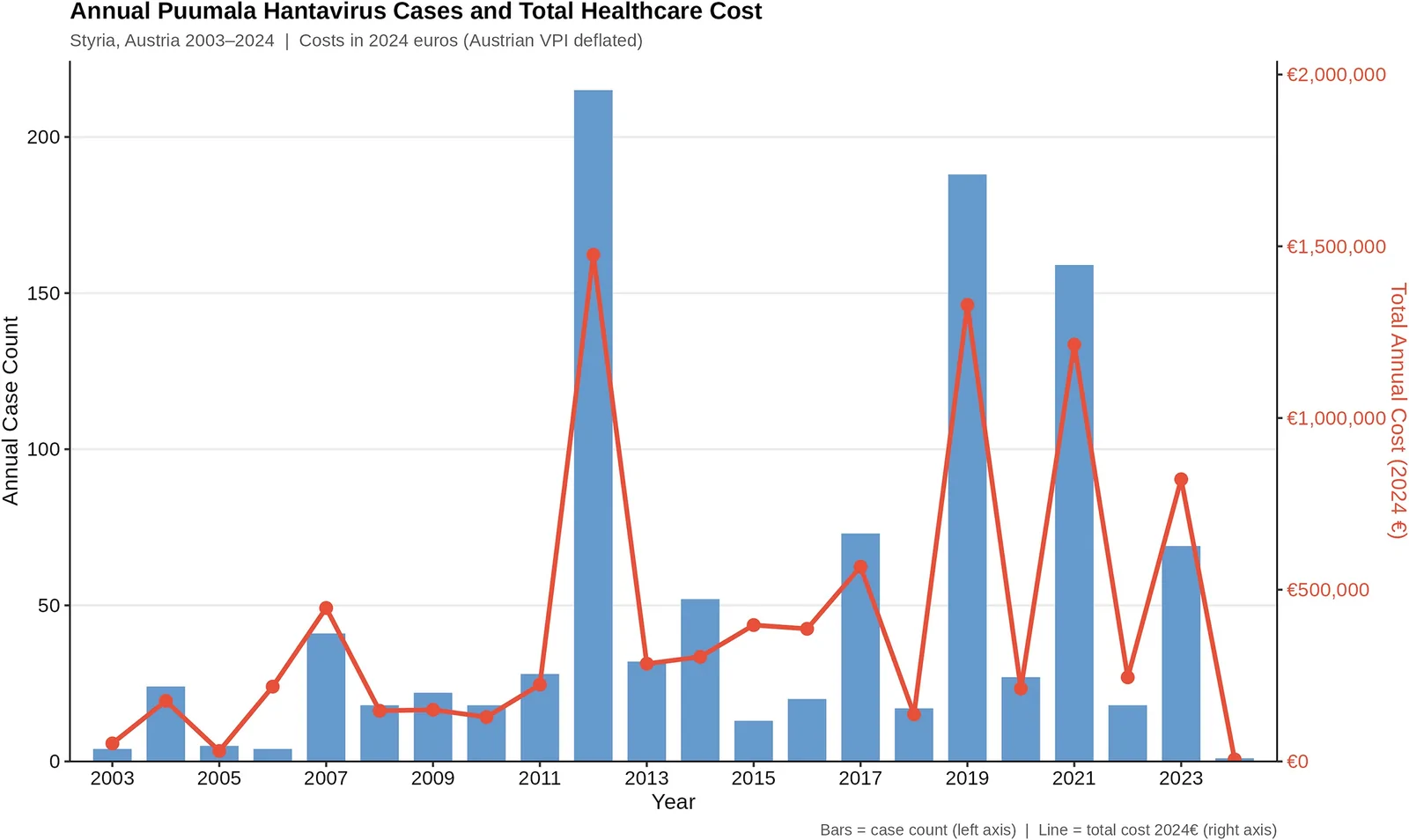
Annual Puumala hantavirus cases and total healthcare cost, Styria 2003–2024. Bars (left axis): annual case count of confirmed PUUV hospitalizations across 20 KAGes hospitals. Line with points (right axis): total annual healthcare cost in 2024-equivalent Euros (Austrian CPI deflated). Note the close tracking between case volume and total cost, and the 3 prominent epidemic years (2012, 2019, and 2021). Partial-year ascertainment applies to 2003 (study start September) and 2024 (study end March).

Total healthcare expenditure closely mirrored case volume, with the epidemic peaks generating €1.93 million (2012), €1.74 million (2019), and €1.56 million (2021) in 2024-equivalent Euros. These 3 years accounted for 562 of 1049 patients (53.6%) and represented a disproportionate share of the cumulative economic burden over the study period.

### Per-case Healthcare Costs

Mean per-case cost across the study period was €9788 (2024-equivalent Euros), with substantial year-to-year variability driven largely by the proportion of severely ill patients requiring ICU care (Figure 2). In high-incidence epidemic years (2012, 2019, and 2021), mean costs ranged between €8000 and €10,000, as severe cases were diluted among many uncomplicated admissions. By contrast, low-incidence years with few ICU admissions produced extreme outliers, such as median per cases costs of €48 294 in 2005 (n = 5, one ICU patient) and €28 970 in 2015 (n = 10).

**Figure 2.**
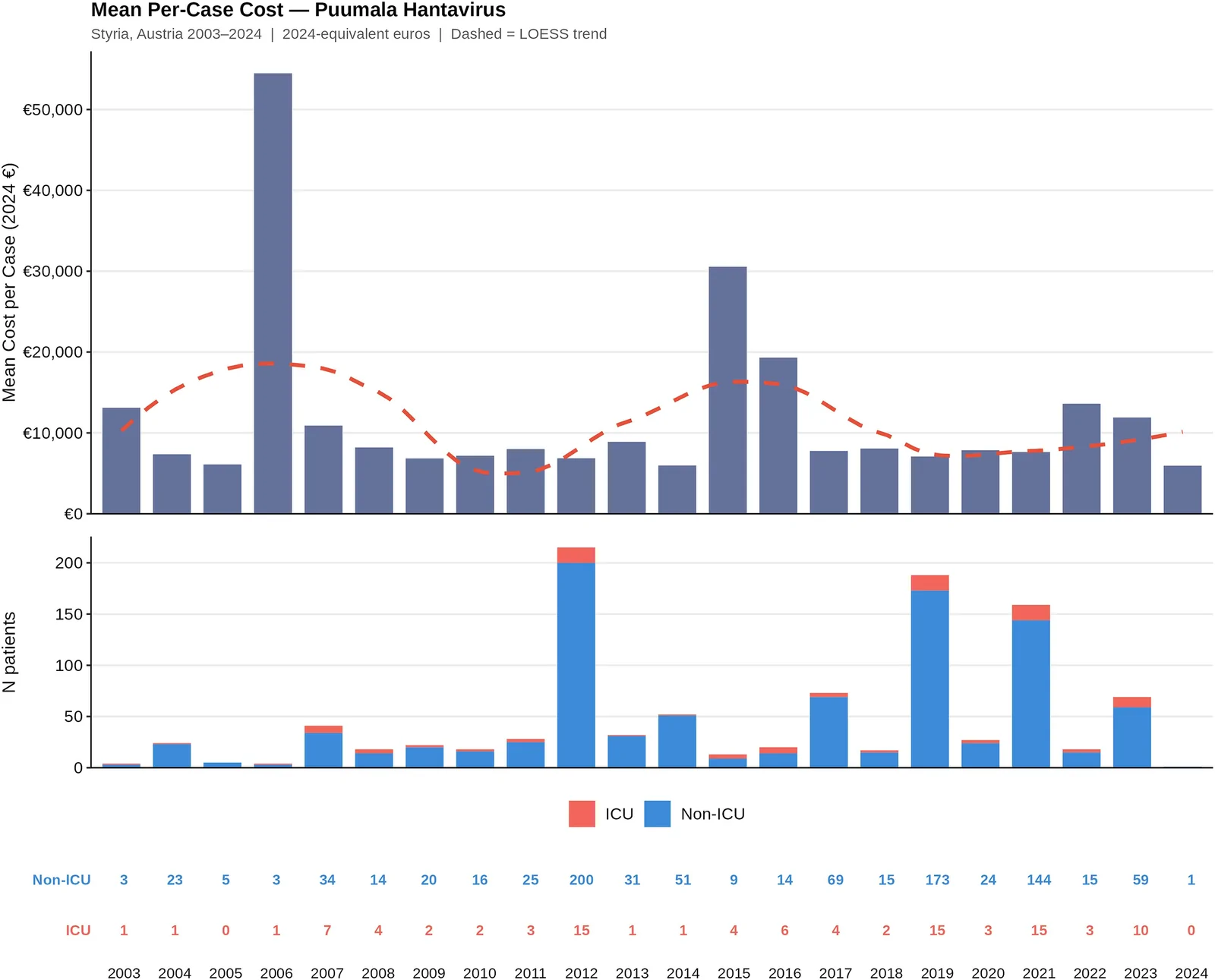
Mean per-case cost by year with ICU/non-ICU breakdown. Upper panel: mean per-case hospitalization cost in 2024-equivalent euros per year (bars) with LOESS trend (dashed red line). Extreme values in low-incidence years (2005 and 2015) reflect the disproportionate influence of individual ICU admissions on annual means when the total case denominator is small. Lower panel: annual case count stratified by ICU and non-ICU admission status. Risk table: absolute counts per group per year.

Cost decomposition showed that base DRG reimbursement dominated total expenditure in epidemic years, whereas ICU daily supplements contributed disproportionately in low-incidence years with clustered severe cases (Supplementary Table 1 and Supplementary Figure 3). Across the cohort, the ICU supplement typically accounted for 12%–25% of annual costs, exceeding 85% in 2006, when 1 patient required 37 ICU care days among only 4 cases.

### Proportion of Styrian Inpatient Healthcare Expenditure

PUUV hospitalization costs represented <0.05% of total annual Styrian inpatient LKF expenditure in most interepidemic years, rising sharply during outbreaks to a study period maximum of 0.274% in 2012 (Figure 3). Secondary peaks occurred in 2019 (0.196%) and 2021 (0.159%), demonstrating that major PUUV epidemics impose a recurrent, measurable burden on the regional public healthcare budget. Total Styrian inpatient LKF expenditure increased from €518 million in 2006 to €1177 million in 2024, reflecting healthcare inflation and system expansion; consequently, PUUV costs represent a declining relative fraction over time despite rising absolute costs in epidemic years.

**Figure 3.**
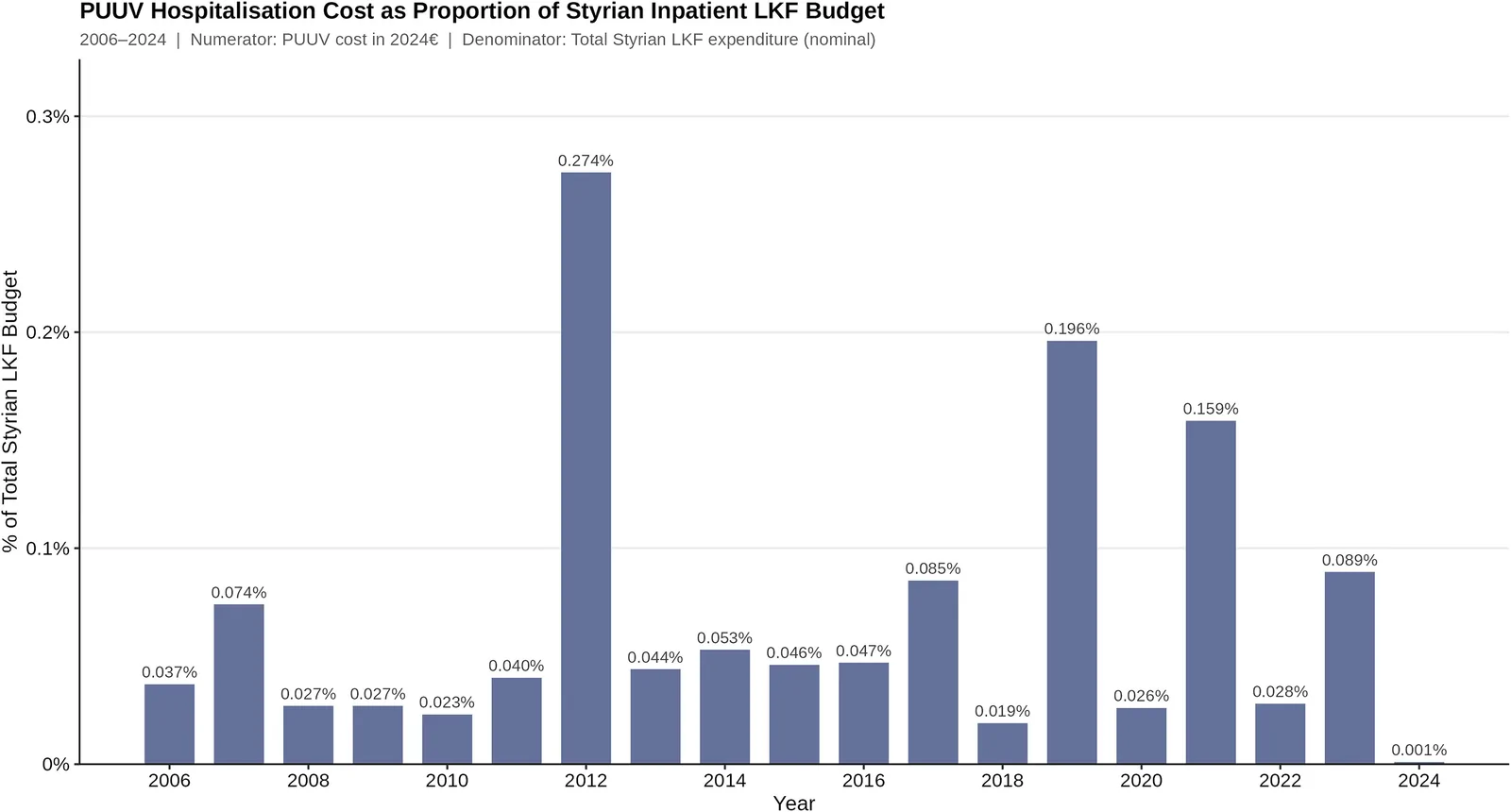
PUUV hospitalization cost as proportion of total Styrian inpatient LKF budget. Annual PUUV hospitalization cost (2024-equivalent Euros) expressed as a percentage of total Styrian inpatient LKF expenditure (nominal Euros; source: Gesundheitsfonds Steiermark). Data available 2006–2024. Peak proportion 0.274% (2012). Note the recurrent budget impact during each of the 3 major epidemic years.

### Hospital Length of Stay-Temporal Trends

LOS declined markedly over the 22-year surveillance period, from 9,5 days in 2003–2006 to 4,5 days in 2023–2024 (Supplementary Table 2 and Supplementary Figure 4). Weighted segmented linear regression identified 2 breakpoints around 2012 and 2021, defining 3 phases of LOS evolution.

In the first phase (2003–2012), LOS fell steeply from 9,5 to 6 days, likely reflecting improved clinical recognition, growing institutional experience, improved diagnostic procedures and refinement of supportive care protocols. The second phase (2012–2021) showed a stabilized median hospital LOS at 6 days, corresponding to an established clinical equilibrium for standard PUUV management. In the third phase (2021–2024) a second decline from 6 to 4,5 days was detected.

Throughout all phases, a persistently wide IQR reflects clinical heterogeneity: prolonged stays in the minority of ICU or renal replacement therapy patients contrast with shorter stays among uncomplicated admissions, progressively lowering the median LOS.

To assess whether early discharge strategies during the inter- and post-coronavirus disease 2019 (COVID-19) pandemic years (2021–2024) were safe, we conducted a non-prespecified analysis of hantavirus-related readmissions to any of the 20 KAGes hospitals and evaluated outpatient follow-up care. Within the KAGes network, we observed no hantavirus-related readmissions within 30 days of discharge from the index hospitalization. However, all patients received at least 1 outpatient follow-up visit, either at an internal medicine clinic or with a general practitioner.

### Admission Predictors of Hospitalization Cost

Multivariable Gamma GLM with MICE imputation (m = 20) identified 2 independent admission-level predictors of 2024-equivalent hospitalization cost across 1049 patients (Supplementary Table 3; Figure 4).

**Figure 4.**
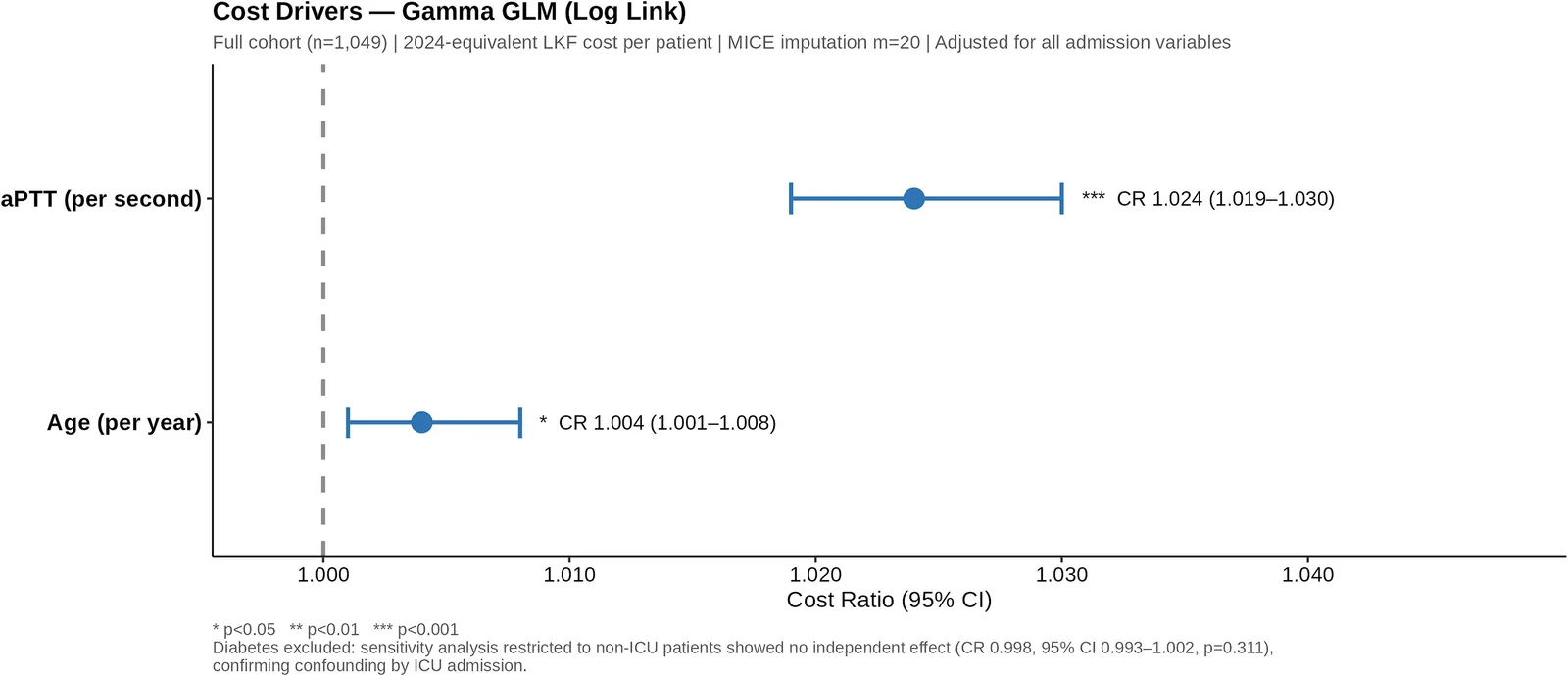
Admission predictors of hospitalization cost—gamma GLM Forest Plot. CR with 95% confidence intervals from multivariable Gamma GLM (log link) analysis of admission predictors of 2024-equivalent LKF hospitalization cost. Full cohort (n = 1049); MICE imputation m = 20; adjusted for all admission variables. Diabetes excluded: sensitivity analysis in non-ICU patients confirmed complete attenuation (CR 0.998, 95% CI .993–1.002, *P* = .311), indicating confounding by ICU severity clustering. aPTT, activated partial thromboplastin time.

Prolonged activated partial thromboplastin time (aPTT) was the strongest predictor: each additional second in the aPTT measured within 24 hours of admission was associated with a 2.4% increase in cost (CR 1.024, 95% CI 1.019–1.030, *P* < .001). This association remained highly significant in non-ICU patients (*P* < .001), indicating that admission coagulopathy is independently associated with cost across the full spectrum of disease. Cost was the only modeled outcome; ICU admission, renal replacement therapy and mortality were not analyzed as endpoints.

Age at admission was the second independent predictor: each additional year increased cost by 0.4% (CR 1.004, 95% CI 1.001–1.008, *P* = .017). Across the cohort, this translates to a ∼20% higher cost for a 70-year-old compared with a 30-year-old patient with otherwise identical characteristics. The effect persisted in non-ICU patients (*P* = .005). Although diabetes mellitus appeared significant in the full-cohort model (CR 1.543, *P* = .001), the effect disappeared in non-ICU patients (CR 0.998, *P* = .311), indicating that the signal was driven by clustering of diabetics among severe ICU cases rather than an independent cost effect. Diabetes was therefore excluded from the final model.

### Deflation Sensitivity Analysis and Cost Differences by Gender

Costs deflated using the OECD Health Expenditure Price Deflator for Austria closely paralleled CPI-deflated costs over the 22-year study period (Supplementary Figure 5). The OECD deflator, reflecting healthcare-sector-specific price changes, produced modestly higher early year estimates (mean +2.5% in 2003–2010) and converged with CPI-based values from 2018 to 2024. The largest difference occurred in 2007 (+6.4%), while estimates were effectively identical from 2018 onward. These results confirm the robustness of the CPI-based deflation approach and indicate that cost estimates are not materially sensitive to the deflation method used.

Unadjusted hospitalization costs were slightly higher in female patients (Wilcoxon *P* = .038), but this was fully explained by their older age distribution (median 50.9 vs 45.5 years). In non-ICU patients, costs were equivalent between sexes (median €7,847, *P* = .874).

## DISCUSSION

This study provides a systematic, longitudinal quantification of the inpatient economic burden of PUUV-related disease in Austria and one of the largest single-region hantavirus cost analyses in Europe. Among 1049 hospitalized patients over 22 years in 20 KAGes hospitals (Styria), the cumulative 2024 equivalent inpatient cost was ∼€10.3 million.

PUUV-related expenditure is sharply episodic. Three epidemic years (2012, 2019, and 2021) accounted for over €5.2 million and more than half of all cases, while interepidemic years rarely exceeded €300,000, sometimes falling below €50 000. The 2012 outbreak (n = 215 cases) solely generated €1.93 million in a single year. This pattern reflects the hyperendemic ecology of PUUV in Styria, presumably driven by multiannual cycles of bank vole (*Myodes glareolus*) populations linked to beech mast. Subsequent peaks in 2019 and 2021 suggest shortening intervals between outbreaks, potentially due to climate-driven changes in mast synchrony or land-use shifts [12, 13].

Although the maximal annual impact (0.274% of total Styrian inpatient LKF expenditure) appears modest, the operational burden is acute: costs arrive over weeks, concentrated in internal medicine and ICUs that cannot easily expand capacity. Against the whole Styrian inpatient budget this share is small, and in interepidemic years almost negligible. The burden is episodic rather than sustained: 3 of 22 years carried 53.6% of all cases and over €5.2 million of the expenditure, arriving within weeks in a few internal medicine wards and ICUs. These findings highlight the importance of integrating PUUV epidemic surveillance, including bank vole monitoring and sentinel rodent seroprevalence, into hospital resource planning, enabling pre-emptive preparation of internal medicine beds and dialysis infrastructure for anticipated epidemic years [14].

Second, aPTT at admission and patient age were the only independent predictors of hospitalization cost, both available within hours of presentation. Each additional second of aPTT prolongation increased cost by 2.4% (CR 1.024, 95% CI 1.019–1.030, *P* < .001), and each additional year of age by 0.4% (CR 1.004, 95% CI 1.001–1.008, *P* = .017). The aPTT effect remained independent of ICU admission in non-ICU patients, demonstrating that coagulopathy drives resource use even in normal ward managed cases, through longer stays, frequent monitoring, and required intensity of care within normal wards [15, 16]. Both variables belong to the routine admission work-up and are reported within 1–2 hours, which makes them useful for anticipating resource use. Cost, however, was the only outcome modeled here; ICU admission, renal replacement therapy, disease severity and mortality were not analyzed and lie outside a cost-of-illness design. Admission aPTT and age are therefore early predictors of resource utilization and not a triage tool, and clinical triage use would require prospective evaluation against clinical endpoints. Only the single admission aPTT entered the model, and laboratory testing generates no separate LKF points, so monitoring intensity cannot explain the association (Supplementary Appendix A2). By contrast, diabetes appeared associated with cost in the full cohort (CR 1.543) but not in non-ICU patients (CR 0.998, *P* = .311), reflecting clustering of a few diabetic patients among severe ICU cases rather than an independent effect. This highlights the importance of prespecified ICU-stratified sensitivity analyses in infectious diseases with bimodal severity, where small clusters of high-cost patients can spuriously influence multivariable models.

Third, median hospital length of stay (LOS) declined by ∼50% over the study period, from 9,5 days in 2003 to 4,5 days in 2023–2024, with segmented regression identifying breakpoints in 2012 and 2021. The initial steep decline (2003–2012) likely reflects improved recognition of PUUV including improved diagnostics, enhanced risk stratification, and accumulation of institutional experience across successive epidemics, including the large 2012 outbreak (n = 215). The subsequent plateau (∼2012–2021) represents consolidation of these management practices. The renewed decline after 2021 coincides with post-COVID-19 hospital bed pressures [17].

Our findings are compatible with, but do not establish, the safety of the shorter hospital stays observed during the inter- and post-COVID-19 years (2021–2024). Despite reduced length of stay, we observed no hantavirus-related readmissions to any of the 20 KAGes hospitals within 30 days after discharge. Importantly, all patients received structured outpatient follow-up, either through internal medicine clinics or general practitioners, which likely contributed to the absence of readmissions. These results are hypothesis-generating: the analysis was not prespecified, covered only readmissions within the KAGes network and within 30 days, and was not designed to demonstrate the safety of shorter stays.

The Austrian LKF DRG system offers several advantages, reflecting actual public payer reimbursement and enabling valid longitudinal comparisons due to stable PUUV DRG assignments and point weights from 2003–2024, anchored by the 2024 tariff of €2.16/point. However, structural limitations exist: inpatient dialysis costs are embedded in the base DRG tariff and are likely underestimated, affecting patients requiring renal replacement therapy. Reimbursed LKF cost also reflects resource use rather than clinical severity, and the per-diem component ties part of it to length of stay. LKF also excludes outpatient follow-up and productivity losses, which may be substantial in this predominantly working-age cohort. Differences in DRG structures and healthcare financing limit generalizability; our findings are directly applicable only to the Austrian system and should be extrapolated with caution [18–21].

To our knowledge, no other comprehensive cost analyses for hantavirus-related disease exist, limiting the ability to contextualize our findings against external data. Consequently, these results provide a unique, system-specific insight into the economic burden of PUUV hospitalizations in Austria.

This study's strengths include its size, duration, and completeness of capture: 1049 patients across 20 hospitals over 22 years, encompassing the full clinical spectrum from uncomplicated admissions to ICU cases severe and adverse course. Use of the LKF DRG system with a legally anchored tariff and stable group assignments enables robust, longitudinal cost assessment, while the 22-year time span, including 3 major epidemics, allows characterization of PUUV's sharply episodic economic impact.

Limitations include underestimation of specific procedural cost like dialysis, extracorporeal membrane oxygenation, exclusion of outpatient follow-up and indirect costs, and readmission ascertainment restricted to the 20 KAGes hospitals and to 30 days after discharge. The small number of severe ICU cases (n = 100) limits subgroup analyses, and partial-year ascertainment in 2003 and 2024, along with low interepidemic case counts, introduces instability in annual per-case estimates [22]. Finally, rare PUUV cases in private hospitals may be missed, though this is expected to be minimal and does not impact the conclusion. The figures reported here should therefore be read as a conservative lower bound of the true economic burden.

In conclusion, PUUV hospitalizations in Styria impose a sharply episodic economic burden, with 3 epidemic years accounting for over half of the total costs during the 22 year study period. Admission aPTT and age are independent early predictors of hospitalization cost and may inform resource planning, but they were not evaluated against clinical outcomes and do not constitute a triage tool. Despite halved median length of stay over 22 years, dialysis, outpatient care, and productivity losses remain underrepresented, highlighting the need for epidemic preparedness and continued surveillance.

## Supplementary Material

ofag607_Supplementary_Data

## Supplementary Data

Supplementary materials are available at *Open Forum Infectious Diseases* online. Consisting of data provided by the authors to benefit the reader, the posted materials are not copyedited and are the sole responsibility of the authors, so questions or comments should be addressed to the corresponding author.
