# Supplementary material for: Epidemiology and Economic Burden of Puumala Virus Infection in Styria, Austria, 2003 to 2024
Source: Open Forum Infect Dis. 2026 Sep 26;13(10):ofag607. doi: 10.1093/ofid/ofag607 (PMC13660694; doi:10.1093/ofid/ofag607)
Supplement: Supplementary file 1 [file ofag607_supplementary_data.docx]

**Supplementary Methods:**

Austrian Hospital Reimbursement System (LKF):

Austrian public hospital financing is governed by the Performance-Oriented Hospital Financing system (Leistungsorientierte Krankenanstaltenfinanzierung, LKF), a diagnosis-related group (DRG) framework administered at the federal level by the Federal Ministry of Social Affairs, Health, Care and Consumer Protection (Bundesministerium für Soziales, Gesundheit, Pflege und Konsumentenschutz, BMSGPK) and implemented at the state level through State Health Funds (Landesgesundheitsfonds). Under this system, each inpatient episode generates a case-level point score composed of a Procedure Component (Leistungskomponente, LK), reflecting diagnostic and therapeutic interventions, and a Per-Diem Component (Tageskomponente, TK), scaled to length of stay relative to the DRG-specific reference range. Point scores are converted to Euros using a state-specific annual cost-covering euro value (kostendeckender Eurowert) negotiated between hospital operators and the Health Fund (Gesundheitsfonds)

For Styrian Krankenanstaltengesellschaft (KAGes) hospitals, the confirmed 2024 tariff was €2.16 per LKF point, as published in Landesgesetzblatt Nr. 125/2023 (Steiermark). This value applies to all KAGes acute care facilities and served as the monetary anchor for the present analysis.

The LKF model undergoes periodic structural revision. Critically, we verified that the DRG group assignment and point weights applicable to PUUV hospitalization remained stable across all model revisions from 2003 to 2024. This stability validated against BMSGPK model documentation for each revision cycle ensures that changes in LKF point scores between years reflect genuine differences in reimbursed resource use rather than artefacts of model restructuring, and permits valid longitudinal comparison of costs across the full study period. We note that the LKF-Modell 2025 introduced a structural shift from 100% to 70% cost coverage per episode; as our study period ends in March 2024, this change does not affect our analysis.

DRG Group Assignment and Point Calculation:

LKF point scores were assigned at the patient level based on the following pre-specified rules derived from the applicable LKF model documentation:

*Base DRG points***:** Three base DRG categories were applied. Adult patients without ICU admission, and adult ICU patients not meeting the criteria for the high-acuity group (see below), were assigned 3,633 points (LK 213, TK 3,420; normal range [Belagsdauermittelwert, BDMW] 6.8 days; lower boundary [Belagsdaueruntergrenze, BDU] 3 days; upper boundary [Belagsdauerobergrenze, BDO] 10 days). Adult patients admitted to the ICU with intubation, hemodynamic compromise as the primary reason for ICU admission, or requirement for extracorporeal membrane oxygenation (ECMO) were assigned 9,305 points (LK 1,873, TK 7,432; BDMW 13.8 days; BDU 7 days; BDO 21 days). Pediatric patients aged under 14 years were assigned 2,449 points (LK 2,436, TK 13; BDMW 2.9 days; BDU 2 days; BDO 4 days).

*ICU daily supplement:* Patients admitted to the ICU received an additive daily supplement reflecting the excess cost of intensive care above standard ward rates. Supplement values were taken from the 2024 LKF model mean (Mittelwert, MW) values as published in the BMSGPK LKF-Modell 2025 documentation, Section 3.2.6. Two ICU intensity classes were applied: IE3 (Intensivbehandlung Stufe 3; 1,872 points/day) for patients with hemodynamic compromise, intubation, or ECMO; and IE2 (Intensivbehandlung Stufe 2; 1,239 points/day) for all other ICU admissions. Daily supplement points were multiplied by the documented ICU length of stay in days and added to the base DRG score.

*Dialysis:* Inpatient renal replacement therapy does not generate additional LKF points beyond the base DRG tariff under the MEL24.xx rule, as the procedure costs are embedded within the case-level reimbursement. Accordingly, no additional points were assigned for hemodialysis or hemofiltration in hospitalized patients.

| **Year** | **N cases** | **Median LOS (days)** | **% ICU** | **% Dialysis** | **Total Cost (2024€)** | **Mean Cost/Case (2024€)** | **% LKF Budget¹** |
| --- | --- | --- | --- | --- | --- | --- | --- |
| **2003** | 3 | 9.0 | 33.3 | 0.0 | €51,408 | €17,136 | N/A |
| **2004** | 4 | 8.5 | 25.0 | 0.0 | €33,228 | €8,307 | N/A |
| **2005** | 5 | 7.0 | 0.0 | 0.0 | €42,012 | €8,402 | N/A |
| **2006** | 4 | 11.0 | 25.0 | 25.0 | €192,234 | €48,059 | N/A |
| **2007** | 41 | 9.0 | 17.1 | 7.3 | €449,658 | €10,967 | 0.071% |
| **2008** | 18 | 8.0 | 22.2 | 11.1 | €200,286 | €11,127 | 0.032% |
| **2009** | 22 | 7.0 | 9.1 | 9.1 | €175,812 | €7,992 | 0.027% |
| **2010** | 18 | 6.0 | 11.1 | 5.6 | €152,712 | €8,484 | 0.023% |
| **2011** | 28 | 7.0 | 10.7 | 3.6 | €274,032 | €9,787 | 0.041% |
| **2012** | 215 | 6.0 | 7.0 | 4.2 | €1,893,888 | €8,809 | 0.272% |
| **2013** | 32 | 6.0 | 3.1 | 3.1 | €269,136 | €8,410 | 0.035% |
| **2014** | 52 | 6.0 | 1.9 | 1.9 | €424,512 | €8,163 | 0.054% |
| **2015** | 10 | 7.5 | 10.0 | 0.0 | €147,528 | €14,753 | 0.018% |
| **2016** | 20 | 6.0 | 5.0 | 5.0 | €152,604 | €7,630 | 0.018% |
| **2017** | 73 | 7.0 | 5.5 | 2.7 | €666,972 | €9,136 | 0.079% |
| **2018** | 17 | 6.0 | 0.0 | 0.0 | €131,868 | €7,757 | 0.015% |
| **2019** | 188 | 6.0 | 8.0 | 4.3 | €1,703,952 | €9,064 | 0.193% |
| **2020** | 27 | 6.0 | 11.1 | 3.7 | €263,736 | €9,768 | 0.027% |
| **2021** | 159 | 6.0 | 9.4 | 5.0 | €1,523,412 | €9,582 | 0.156% |
| **2022** | 18 | 5.0 | 16.7 | 0.0 | €218,376 | €12,132 | 0.022% |
| **2023** | 69 | 5.0 | 14.5 | 5.8 | €962,028 | €13,942 | 0.089% |
| **2024** | 1 | 4.0 | 0.0 | 0.0 | €7,848 | €7,848 | 0.001% |

**Supplementary Table 1:** **Annual Economic Summary (2003–2024)**

Annual case counts, clinical outcomes, and 2024-equivalent hospitalisation costs for Puumala virus infections in Styrian public hospitals. Costs were calculated using the LKF point system and deflated to 2024 euros using the Austrian consumer price index (Verbraucherpreisindex, VPI; base 2020 = 100).

¹ Proportion of total annual Styrian inpatient LKF expenditure. Source: Gesundheitsfonds Steiermark / KAGes. Data available from 2006 onwards; 2003–2005 not available (N/A), because consolidated reporting by the Gesundheitsfonds Steiermark began when the state health funds were established in the 2005 Austrian health care reform.

Abbreviations: LOS = length of stay; ICU = intensive care unit; LKF = Leistungsorientierte Kran

kenanstaltenfinanzierung

| **Segment** | **Period** | **Slope (days/year)** | **Breakpoint (est. year)** | **Clinical interpretation** | **p** |
| --- | --- | --- | --- | --- | --- |
| **1** | 2003 – ~2012 | −1.2 | ~2012 | Rapid decline: improved recognition & management | <0.001 |
| **2** | ~2012 – ~2021 | −0.1 | ~2021 | Plateau: equilibrium in standard PUUV care at ~6 days | 0.412 |
| **3** | ~2021 – 2024 | −0.6 | N/A | Renewed decline: post-COVID discharge pressure & ambulatory follow-up improved recognition & management | 0.031 |

**Supplementary Table 2: Weighted segmented linear regression of annual median hospital length of stay (LOS).** Each year was weighted by the number of cases. Two statistically significant breakpoints were identified using the R package *segmented* (npsi = 2, n.boot = 100). Approximate slope values are shown; exact estimates, confidence intervals, and p-values are available from the segmented model output.

Base model: lm(los_median ~ year, weights = n_cases). Breakpoint years were estimated with confidence intervals via slope() and confint() in the *segmented* package. Slope p-values reflect the significance of the linear trend within each segment

| **Variable** | **Cost Ratio** | **95% CI** | **p-value** | **Sig.** |
| --- | --- | --- | --- | --- |
| **aPTT (per second)** | 1.024 | 1.019 – 1.030 | <0.001 | ******* |
| **Age (per year)** | 1.004 | 1.001 – 1.008 | 0.017 | ***** |
| Diabetes² | 1.543† | 1.195 – 1.992 | 0.001 | *** |

**Supplementary Table 3:** **Multivariable Gamma generalized linear model with log link.**

Outcome: 2024-equivalent LKF cost per patient. Predictors: all admission variables with <15% missing data. Missing values were imputed using multiple imputation by chained equations (MICE; m=20; predictive mean matching for continuous, logistic regression for binary variables). Results were pooled using Rubin’s rules.

* p<0.05, ** p<0.01, *** p<0.001. Cost ratio (CR) represents the multiplicative change in mean cost per unit increase.

² Diabetes was significant in the full cohort (CR 1.543, 95% CI 1.195–1.992, p=0.001) but not in non-ICU patients (CR 0.998, 95% CI 0.993–1.002, p=0.311), indicating confounding by clustering of diabetic patients among severe ICU cases. Diabetes is greyed out and excluded from the final model.

Variables excluded a priori due to >15% missing: height, weight, temperature, IL-6, procalcitonin, NT-proBNP, troponin T, D-dimer, antithrombin III, fibrinogen, ferritin, triglycerides.

Abbreviations: aPTT = activated partial thromboplastin time; MICE = multiple imputation by chained equations

**Supplementary Figure 1. Study flow diagram for the economic burden analysis of PUUV hospitalization**, Styria, Austria (September 2003 – March 2024). Of 1,064 records screened, 15 patients were excluded due to missing data, yielding a final cohort of *n* = 1,049 across 20 hospitals. Inpatient costs were estimated using the Austrian LKF system and deflated to 2024 euros via the CPI. Total cumulative cost was €10.26 million (mean €9,788/case; median €7,848/case). Independent predictors of cost were aPTT and age. apTT -activated partial thromboplastin time; CPI – consumer price index; DRG – diagnosis related group; ICU – intensive care unit; ICU level II - admission without intubation, hemodynamic instability, or ECMO ICU level III - intubation, hemodynamic instability, or ECMO; LOS - Length of stay;

**Supplementary Figure 2. Map of Styria showing PUUV cases per district**. Color shading indicates case counts, with the highest numbers observed in the Eastern and Southern regions of the state.

**Supplementary Figure 3: Total Annual Healthcare Cost Decomposed by Cost Component**

Total annual PUUV hospitalization cost (2024-equivalent euros) decomposed into base DRG component (blue) and ICU daily supplement component (red), Styria 2003–2024. Base DRG cost dominates in all high-incidence years. The ICU supplement fraction is proportionally largest in low-incidence years with coincidental severe cases (2006, 2015–2016, 2022).

**Supplementary Figure 4. Hospital Length of Stay - Segmented Regression Analysis**

Weighted segmented linear regression of annual median LOS (points sized by annual case count; IQR ribbon). Red line: segmented fit. Blue dashed vertical lines: estimated breakpoints (~2012, ~2021) defining three clinical phases of LOS evolution. Weights proportional to annual case count. Styria, Austria 2003–2024.

**Supplementary Figure 5. Sensitivity Analysis - Deflation Method Comparison**

Total annual PUUV hospitalization cost expressed in 2024-equivalent euros using two deflation methods: Austrian VPI/CPI (Statistik Austria; blue) and OECD Health Expenditure Price Deflator for Austria (red). The close tracking of both lines across all years confirms the robustness of the primary VPI-based deflation approach. Maximal divergence in the early study period does not exceed 6.4% in relative terms.

**Alt text section:**

**Supplementary Figure 1 (Flow diagram).** Flow diagram showing the study selection process for the economic burden analysis of Puumala hantavirus (PUUV) hospitalizations in Styria, Austria (September 2003 – March 2024). Of 1,064 records screened, 15 were excluded due to missing data, yielding a final analytic cohort of 1,049 patients across 20 hospitals. The diagram illustrates the LKF-based cost estimation pathway, stratified by ICU level (level II: no intubation, hemodynamic instability, or ECMO; level III: intubation, hemodynamic instability, or ECMO) and non-ICU admission. Total cumulative cost was €10.26 million (mean €9,788 per case; median €7,848 per case). Independent cost predictors identified were aPTT and age at admission. Abbreviations: aPTT, activated partial thromboplastin time; CPI, consumer price index; DRG, diagnosis-related group; ECMO, extracorporeal membrane oxygenation; ICU, intensive care unit; LKF, Leistungsorientierte Krankenanstaltenfinanzierung; LOS, length of stay.

**Supplementary Figure 2.** Choropleth map of Styria, Austria, displaying confirmed PUUV case counts by district across the study period (2003–2024). Color intensity reflects the number of cases per district, with the highest case burdens concentrated in the eastern and southern regions of the state.

**Supplementary Figure 3.** Stacked bar graph of total annual PUUV hospitalization cost (2024-equivalent euros) decomposed into base DRG component (blue) and ICU daily supplement component (red), Styria, Austria, 2003–2024. Base DRG cost dominates expenditure in all high-incidence years. The ICU supplement fraction is proportionally largest in low-incidence years coinciding with sporadic severe cases (2006, 2015–2016, 2022).

**Supplementary Figure 4.** Weighted segmented linear regression of annual median hospital length of stay (LOS) for PUUV hospitalizations, Styria, Austria, 2003–2024. Data points are sized proportionally to annual case count; the interquartile range is shown as a shaded ribbon. The red line represents the segmented regression fit; blue dashed vertical lines indicate estimated breakpoints at approximately 2012 and 2021, defining three distinct clinical phases of LOS evolution.

**Supplementary Figure 5.** Line graph comparing total annual PUUV hospitalization cost (2024-equivalent euros) derived from two deflation methods: the Austrian consumer price index (VPI/CPI; Statistik Austria; blue) and the OECD Health Expenditure Price Deflator for Austria (red), 2003–2024. Both series track closely across all years, with a maximum relative divergence of 6.4% in the early study period, confirming the robustness of the primary VPI-based deflation approach.

**Supplementary Appendix:**

**A1. Hospital financing and where the cost of PUUV hospitalization falls**

Austrian acute inpatient care is financed collectively rather than billed to the individual patient. Statutory social health insurance contributions are pooled with federal, state and municipal tax revenue in the state health funds (Landesgesundheitsfonds), and hospital operators are reimbursed from this pool on the basis of LKF points. The expenditure reported in this study is therefore borne by the public payer, and ultimately by insured persons and taxpayers, while patients are liable only for a small statutory daily co-payment and individual hospitals carry no case-specific financial exposure. Coverage is close to universal, so the analysis is not affected by insurance status.

**A2. What LKF cost does and does not measure**

LKF points measure reimbursed resource utilization and are not a validated measure of clinical severity. The per-diem component scales with length of stay, and documentation and coding practices may vary between institutions and over time, so a higher cost should be read as greater reimbursed resource use rather than as more severe disease. Laboratory investigations, including serial coagulation monitoring, also generate no separate LKF points: case-level cost in this study is composed of the base DRG point score and, where applicable, the ICU per-diem supplement, as set out above. The intensity of laboratory monitoring can therefore not in itself increase the costs reported here, and only the single aPTT value obtained within 24 hours of admission entered the cost model. Whether serial coagulation monitoring alters clinical management in PUUV infection was not assessed in this study.

**A3. Case ascertainment period**

The study period was determined by the reach of retrievable records rather than by design. Electronic laboratory and administrative hospital records within the KAGes network begin in September 2003; PUUV diagnoses made before that date are held in paper archives only and could not be identified in a systematic and verifiable manner. Data collection was closed in March 2024. The first and last calendar years of the study period are therefore incomplete (September to December 2003 and January to March 2024) and are reported as partial years throughout.

**A4. Availability of Styrian inpatient budget data**

Consolidated Styrian inpatient LKF expenditure has been reported by the Gesundheitsfonds Steiermark only since the state health funds were established under the 2005 reform of the organization and financing of Austrian health care (Art. 15a B-VG agreement), the first full reporting year being 2006. Comparable aggregate figures for 2003 to 2005 are not published and could not be obtained from KAGes or from the Gesundheitsfonds Steiermark. The proportional analysis is accordingly restricted to 2006 to 2024.
